# Exploring the Regulatory Role of XIST-microRNAs/mRNA Network in Circulating CD4^+^ T Cells of Hepatocellular Carcinoma Patients

**DOI:** 10.3390/biomedicines11071848

**Published:** 2023-06-27

**Authors:** Lien-Hung Huang, Cheng-Shyuan Rau, Yueh-Wei Liu, Chia-Jung Wu, Peng-Chen Chien, Hui-Ping Lin, Yi-Chan Wu, Chun-Ying Huang, Ting-Min Hsieh, Ching-Hua Hsieh

**Affiliations:** 1 Department of Neurosurgery, Kaohsiung Chang Gung Memorial Hospital, Chang Gung University College of Medicine, Kaohsiung 833, Taiwan; ahonbob@gmail.com (L.-H.H.); ersh2127@cloud.cgmh.org.tw (C.-S.R.); 2 Department of General Surgery, Kaohsiung Chang Gung Memorial Hospital, Chang Gung University College of Medicine, Kaohsiung 833, Taiwan; anthony0612@adm.cgmh.org.tw; 3 Department of Trauma Surgery, Kaohsiung Chang Gung Memorial Hospital, Chang Gung University College of Medicine, Kaohsiung 833, Taiwan; alice8818@yahoo.com.tw (C.-J.W.); venu_chien@hotmail.com (P.-C.C.); poppy952@gmail.com (H.-P.L.); janewu0922@gmail.com (Y.-C.W.); junyinhaung@cgmh.org.tw (C.-Y.H.)

**Keywords:** hepatocellular carcinoma (HCC), CD4^+^ T cells, next generation sequencing (NGS), long non-coding RNAs (lncRNAs), microRNAs (miRNAs)

## Abstract

Hepatocellular carcinoma (HCC) is one of the most common cancers and the main cause of cancer-related death globally. Immune dysregulation of CD4^+^ T cells has been identified to play a role in the development of HCC. Nevertheless, the underlying molecular pathways of CD4^+^ T cells in HCC are not completely known. Thus, a better understanding of the dysregulation of the lncRNA-miRNA/mRNA network may yield novel insights into the etiology or progression of HCC. In this study, circulating CD4^+^ T cells were isolated from the whole blood of 10 healthy controls and 10 HCC patients for the next-generation sequencing of the expression of lncRNAs, miRNAs, and mRNAs. Our data showed that there were different expressions of 34 transcripts (2 lncRNAs, XISTs, and MIR222HGs; 29 mRNAs; and 3 other types of RNA) and 13 miRNAs in the circulating CD4^+^ T cells of HCC patients. The expression of lncRNA-XIST-related miRNAs and their target mRNAs was confirmed using real-time quantitative polymerase chain reaction (qPCR) on samples from 100 healthy controls and 60 HCC patients. The lncRNA–miRNA/mRNA regulation network was created using interaction data generated from ENCORI and revealed there are positive correlations in the infiltration of total CD4^+^ T cells, particularly resting memory CD4^+^ T cells, and negative correlations in the infiltration of Th1 CD4^+^ T cells.

## 1. Introduction

Hepatocellular carcinoma (HCC) is the most prevalent type of liver cancer, accounting for 90% of all occurrences of primary liver cancer [1]. HCC is a recognized inflammation-related cancer form. The immunological microenvironment of the liver plays a crucial role in the etiology of illness [2]. Infection with the hepatitis B virus or the hepatitis C virus [3], persistent alcohol consumption, nonalcoholic steatohepatitis, and exposure to aflatoxin B are all variables that might increase the likelihood of developing hepatocellular carcinoma [4]. When certain infectious pathogens generate persistent inflammation, innate and adaptive immune system modifications influence disease development [5]. Persistent inflammation promotes the development of HCC by the infiltration of diverse immune cells, including dendritic cells, macrophages, natural killer cells, neutrophils, T cells, and B cells [6]. The exploration of the mediating pathway may help the understanding of cancer progression [7,8,9] and create a rational approach for effective cancer therapy [10].

T cells are recognized to have a crucial role in the development of HCC. Among the subtypes of T cells, the dysregulation of the function of CD4^+^ T cells is emerging as a factor in HCC. By secreting cytokines and activating CD8^+^ T lymphocytes, CD4^+^ T cells play a role in antitumor immune responses [11]. Activated CD4^+^ T cells stimulate CD8^+^ cytotoxic T lymphocytes (CTLs) via dendritic cells or by directly secreting IL-2. After receiving signals from dendritic cells, CD4^+^ T cells are activated and polarized to the Th1 phenotype, producing effector cytokines such as interferon-gamma (IFN-γ) and tumor necrosis factor-alpha (TNF-α). CD4^+^ T cells also stimulate the differentiation and maturation of B cells into plasma cells [12]. After specific cytokine signaling and transcription factor expression, CD4^+^ T cells may differentiate into several subsets, including T-helper 1 (Th1), T-helper 2 (Th2), T-helper 17 (Th17), follicular helper T (Tfh), and T-regulatory (Tregs) cells [13]. The steady decline in CD4^+^ T lymphocytes is substantially related to increased mortality and a reduced survival time in patients with HCC [14].

Long noncoding RNAs (lncRNAs) play a significant role in a variety of physiological and pathological processes via several regulatory mechanisms [15]. Many pieces of evidence point to the fact that the abnormal expression of certain lncRNAs in HCC contributes to the progression of the illness [16,17,18]. Several studies have demonstrated that lncRNAs are essential for T cell development, activation, and pathogen response [19,20,21]. Accumulating evidence indicates that lncRNA and microRNA (miRNA) interact to control gene expression [22,23]. LncRNAs may act as miRNA sponges or bind to sites on the target genes of miRNAs to reduce the interaction of miRNA and its target mRNA. On the other hand, miRNAs can regulate the stability and half-life of lncRNA by target lncRNA. In addition, some lncRNAs containing miRNA sequences can be cleaved by Dicer and/or Drosha to produce miRNAs [24].

There has been no extensive research on the lncRNA–miRNA/mRNA network of circulating CD4^+^ T cells in HCC. Under the hypothesis that circulating CD4^+^ T cells in HCC have a different lncRNA–miRNA/mRNA network than that in normal patients, in this work, circulating CD4^+^ cells from HCC clinical samples were extracted and compared to those from healthy people to illustrate the expression landscape of lncRNAs, miRNAs, and mRNAs, as well as the regulatory network of miRNA–mRNA/lncRNA.

## 2. Materials and Methods

### 2.1. Study Patient Population

This study was approved by the Institutional Review Board of Chang Gung Memorial Hospital (IRB number: 201900911B0), and all participants provided written informed consent. Blood samples were taken from patients with or without HCC. We employed 10 healthy controls and 10 HCC patients in our NGS study. The demographics of these twenty people are presented in Supplementary Table S1. To validate the expression of discovered genes, 100 samples of healthy controls and 60 samples of HCC, including 24 stage 1, 28 stage 2, and 8 stage 3 patients, were utilized. The demographics of these individuals from the validation cohort are given in Supplementary Table S2.

### 2.2. Isolation of Circulating CD4^+^ Cells and RNA Extraction

PBMC were isolated using density gradient centrifugation and Ficoll-PaqueTM PREMIUM solution (GE Healthcare, 17544202). Anti-human CD4 Particles—DM magnetic nanoparticles (BD IMagTM, 557767)—were used to isolate CD4^+^ cells according to the manufacturer’s procedure. The collected CD4^+^ cells were lysed using 700 uL of QIAzol Lysis Reagent, and RNA was extracted using an miRNeasy Mini Kit (217004, QIAGEN, Hilden, North Rhine-Westphalia, Germany) per the manufacturer’s instructions. Then, 50 l RNase-free water was added to the RNA-isolated samples.

### 2.3. RNA Quality Determination and NGS

The extracted RNA samples were quantified using a NanoDrop 2000 (Thermo Scientific, Waltham, MA, USA) spectrophotometer and a Qubit 2.0 fluorometer (Thermo Scientific). Qubit/NanoDrop ratio (1 ± 0.3 assigned as well) and Caliper RNA Quality Score (RQS) adopting Caliper LabChip GX Electrophoresis System were utilized to evaluate RNA quality (Caliper Life Sciences, Hopkinton, MA, USA). Using the TruSeq^®^ Stranded Total RNA Sample Preparation Instructions, cDNA libraries were constructed (15031048, Illumina, San Diego, CA, USA). Equal samples from each library were pooled and sequenced with a NextSeq 500 (Illumina).

For RNA-Seq, the row sequences were assessed for quality using FastQC v0.11.8. and trimmed for primer–adaptor sequences using an RNA-seq alignment tool from BaseSpace (Illumina), followed by alignment to the human reference genome (hg38) with STAR v2.7.3a. The expression levels and differential expression analysis were estimated using DESeq2 v1.24.0. (selection criteria: adjusted *p* value of < 0.05 and |fold change| > 2). 

For miRNA-Seq, the sequences were assessed for quality and adapter trimming using Trim_Galore v. 0.4.4, followed by alignment to the human reference genome (hg38) from UCSC with bwa v. 0.7.15. The expression profiles were produced using BCGSC miRNA Profiling Pipeline, and differential expression analysis was estimated using DEseq2 v. 1.16.1.

### 2.4. GO and KEGG Enrichment Analysis

Gene Ontology (GO) and the Kyoto Encyclopedia of Genes and Genomes (KEGG) enrichment analysis were performed using the R package clusterProfiler (version 3.18.1) to detect the enrichment of signaling pathways. GO analysis included analyses of biological processes, cellular components, and molecular functions. GO and KEGG terms with *p* < 0.05 were considered significantly enriched by differentially expressed genes (DEGs).

### 2.5. Real-Time Quantitative Polymerase Chain Reaction

For measuring the expression of mRNAs and lncRNAs, the transcripts were reverse-transcribed to cDNA using High-Capacity cDNA Reverse Transcription Kit (4368814, Applied Biosystems, Waltham, MA, USA). A 7500 Real-Time PCR System and a Power SYBR Green PCR Master Mix (ABI 4367659) were utilized for gene expression investigation (Applied Biosystems). The sequences of the primers used are shown in Supplementary Table S3.

For miRNA, the transcripts were reverse-transcribed to cDNA using TaqMan™ Advanced miRNA cDNA Synthesis Kit (Applied Biosystems, A28007). TaqMan Advanced miRNA Assay (Applied Biosystems, A25576) and TaqMan Fast Advanced Master Mix were utilized for the miRNA expression study (Applied Biosystems, 444557).

### 2.6. The Interaction of miRNA-lncRNA and miRNA–mRNA

The Encyclopedia of RNA Interactomes (ENCORI; https://starbase.sysu.edu.cn/; vers. 3.0, accessed on 1 April 2023) was used to obtain information on the miRNA-lncRNA or miRNA–mRNA interaction [25]. ENCORI is an open-source platform for researching the interactions between miRNAs and their targets. At least three miRNA-target prediction databases, including PITA, miRmap, and TargetScan, were utilized to identify the targeted miRNAs of candidate genes in the present work.

### 2.7. CD4^+^ T Cells Infiltration Analysis

The association between CD4^+^ T cells infiltrates and differential express genes was evaluated using TIMER2.0 website (http://timer.cistrome.org/, accessed on 1 April 2023), which provides robust estimation of immune infiltration levels using website algorithms, including TIMER, EPIC, xCell, CIBERSORT, and quanTIseq [26].

### 2.8. Statistical Analysis

The Windows version of GraphPad Prism 5 was used for all statistical tests (version 5.01). The data were provided as a mean ± standard error (SEM). The Mann–Whitney U test was used to conduct the pairwise comparisons, and the resulting *p* values were then shown. A value of *p* 0.05 was used to indicate statistical significance in all two-tailed tests.

## 3. Results

### 3.1. NGS Analysis

**Figure 1:**
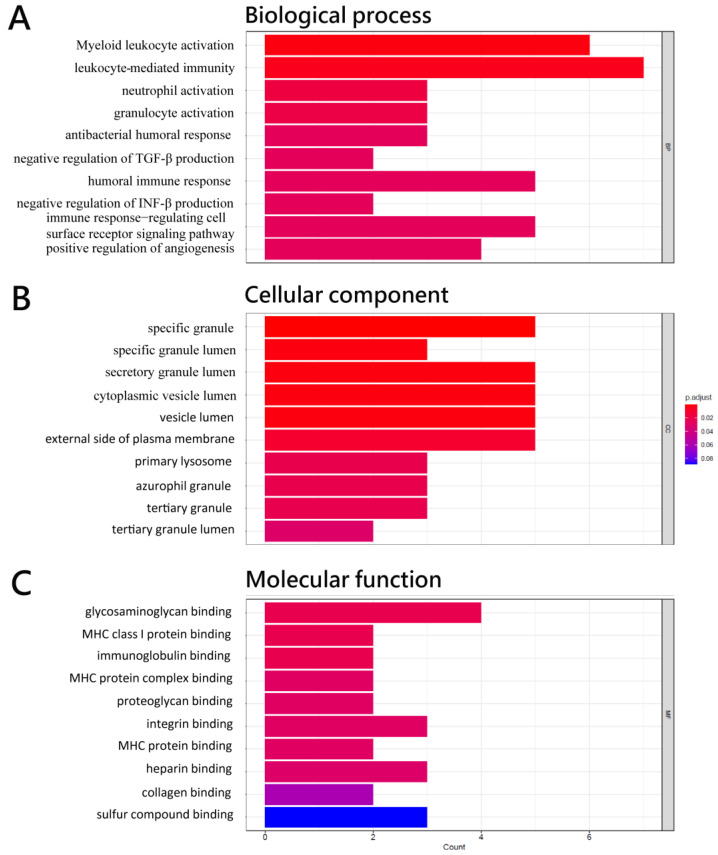
Functional enrichment analysis of differentially expressed genes in CD4^+^ T cells of HCC with the GO terms of (**A**) biological process, (**B**) cellular component, and (**C**) molecular function of 34 differentially expressed genes.

**Figure 2:**
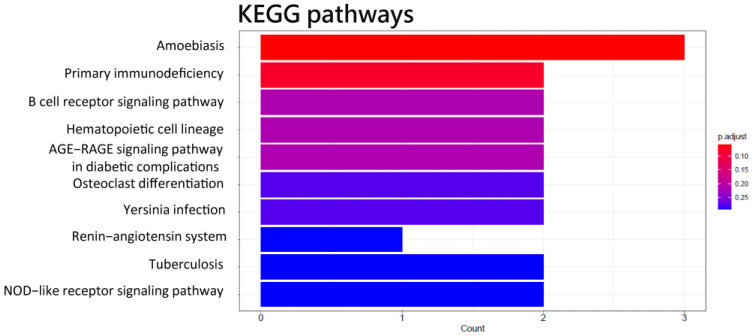
KEGG pathways of 34 differentially expressed genes in the functional enrichment analysis of differentially expressed genes in CD4^+^ T cells of HCC.

RNA-Seq analysis of circulating CD4^+^ cells from 10 HCC patients vs. 10 health control showed that the expression of 34 transcripts was significantly differentially expressed in circulating CD4^+^ T cells. A total of 2 of the 34 transcripts were lncRNAs (XIST and MIR222HG), 29 were mRNAs, and 3 were of other types of RNAs (Table 1). Among these two lncRNAs, MIR222HG was up-regulated and XIST was down-regulated in HCC patients. Among the 29 mRNAs, 12 were up-regulated and 17 were down-regulated in HCC patients (Table 1). GO analysis revealed that the biological processes of 34 differentially expressed genes (DEGs) were predominantly associated with leukocyte-mediated immunity, myeloid leukocyte activation, neutrophil activation, granulocyte activation, and humoral immune response (Figure 1A). The cellular component of these DEGs was engaged in granule lumens and vesicle lumens (Figure 1B). The molecular function of these DEGs was involved in glycosaminoglycan binding, immunoglobulin binding, and MHC protein complex binding (Figure 1C). The results of KEGG pathway enrichment analysis showed that DEGs were mostly implicated in the primary immunodeficiency and B cell receptor signaling pathways (Figure 2).

**Figure 3:**
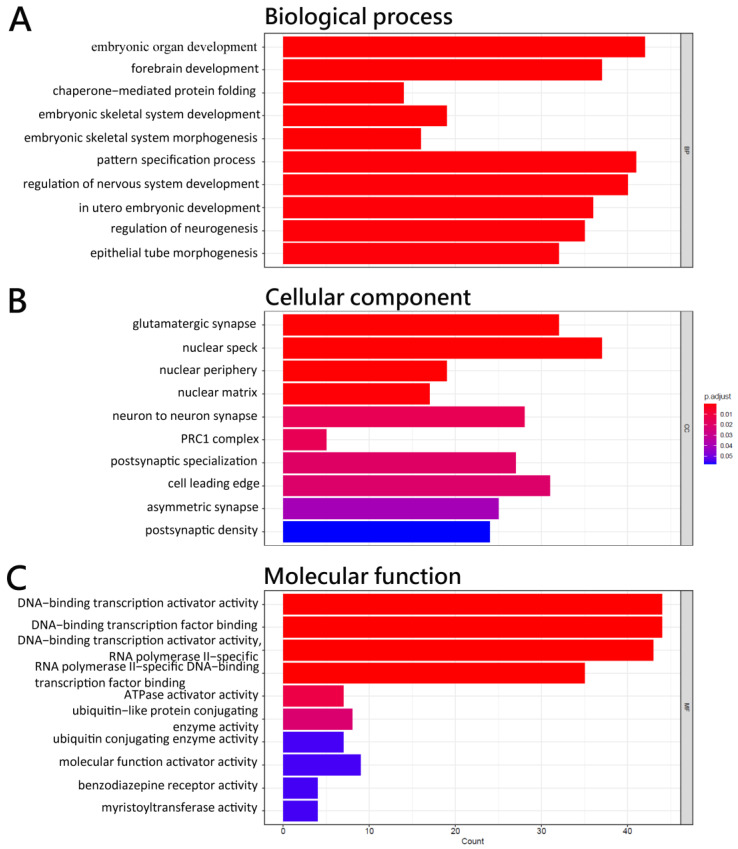
Functional enrichment analysis of predicted target genes of differentially expressed miRNA in CD4^+^ T cells of HCC with the GO terms of (**A**) biological process, (**B**) cellular component, and (**C**) molecular function of predicted target genes of differentially expressed miRNAs.

**Figure 4:**
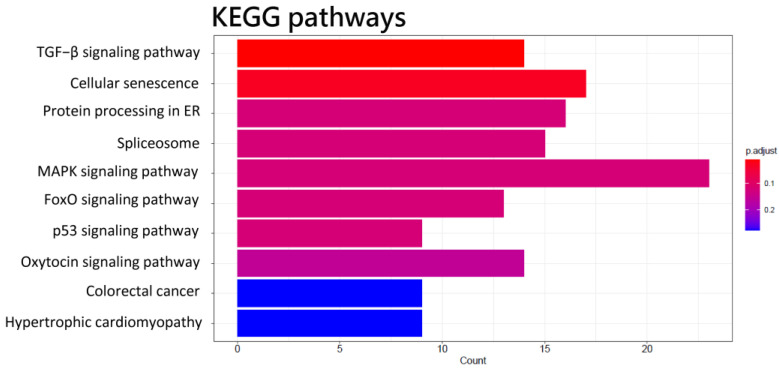
KEGG pathways of predicted target genes of differentially expressed miRNAs in the functional enrichment analysis of differentially expressed genes in CD4^+^ T cells of HCC.

The miRNA-seq study revealed that the expression of 13 miRNAs, 1 of which was up-regulated and 12 of which were down-regulated, was significantly different between HCC patients and healthy controls (Table 2). The predicted target genes of these miRNAs with differential expression are reported in Table 3. GO analysis revealed that these miRNA target genes are crucial to protein activity in tissue development in biological functions (Figure 3A), synapse structure in cellular components, and DNA-binding transcription activators in molecular functions (Figure 3C). The KEGG pathway enrichment result for these predicted target genes was involved in the TGF−β signaling pathway, cellular senescence, protein processing in the endoplasmic reticulum, spliceosome, and several signaling pathways (Figure 4).

### 3.2. The miRNA-lncRNA or miRNA–mRNA Interaction by ENCORI

ENCORI was used to study the interaction between miRNA and lncRNA as well as miRNA and mRNA. Based on ENCORI analysis, there are 8 of 13 differential expressions of miRNAs to XIST (Table 3), including hsa-miR-34a-5p, hsa-miR-369-3p, hsa-miR-370-3p, hsa-miR-494-3p, hsa-miR-495-3p, hsa-miR-543, hsa-miR-654-3p, and hsa-miR-889-3p. However, there is no differential expression of miRNAs related to MIR222HG. The target mRNAs of eight XIST-related miRNAs predicted by ENCORI were identified (Supplementary Table S4) and compared to their expression levels from the DEGs in this study (Table 4). Due to the limited number of isolated CD4^+^ T cells, only those up-regulated mRNAs that are related to down-regulated miRNAs were chosen for further real-time qPCR validation in this work. Five mRNAs were identified: an inhibitor of DNA binding 1 (ID1), Interleukin 1 beta (IL1β), Annexin A3 (ANXA3), Period circadian regulator 1 (PER1), and Fibronectin 1 (FN1).

### 3.3. Validation of the Expression of Transcripts

**Figure 5:**
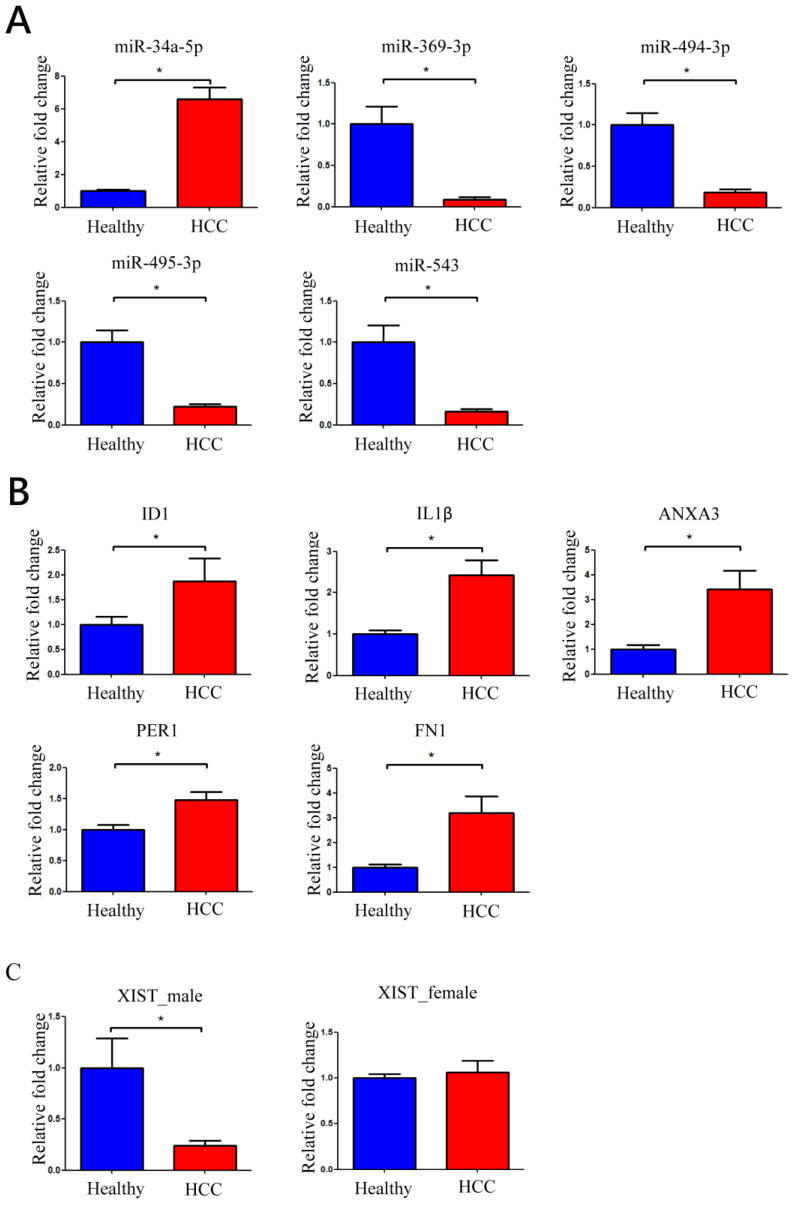
miRNA/mRNA/lncRNA expression level verification. Quantification of (**A**) miRNAs, (**B**) differentially expressed miRNA-targeted mRNAs, and (**C**) XIST expression using real-time qPCR. * indicated *p* < 0.05 in the comparison between healthy patients and those with HCC.

Real-time qPCR was used to determine the expression levels of XIST, 8 XIST-related miRNAs, and their target mRNAs in 160 validation samples (100 health control and 60 HCC samples). Figure 5A demonstrates that miR-34a-5p was substantially up-regulated, while miR-369-3p, miR-494-3p, miR-495-3p, and miR-543 were significantly down-regulated in HCC patients using real-time qPCR. These results are consistent with those disclosed by NGS. Nevertheless, the expression levels of three (miR-370-3p, miR-654-3p, and miR-889-3p) of the eight miRNAs associated with XIST were either modest or undetectable.

We then measured the identified potential target mRNAs utilizing real-time qPCR. These mRNAs were chosen based on the intersection of the target mRNAs of the aforementioned substantially down-regulated miRNAs and those presenting increased expression in the NGS experiments. Five mRNAs (ID1, IL1β, ANXA3, PER1, and FN1) were selected for the real-time qPCR experiment. ID1, which is a target for miR-494-3p, miR-495-3p, miR-654-3p, and miR-889-3p, was significantly up-regulated in HCC patients. IL1β, as a target for miR-34a-5p and miR-495-3p; ANXA3, as a target of miR-369-3p; PER1, as a target for miR-34a-5p and miR-370-3p; and FN1, as a target for miR-543, had significant up-regulation in HCC patients than healthy subjects (Figure 5B).

Given that XIST is a sex-linked lncRNA, the amounts of XIST expression for males and females were detected separately using real-time qPCR analyses. XIST expression was considerably suppressed in male HCC patients but not in female HCC patients (Figure 5C). In this study, the expression levels of XIST-related miRNAs and their regulated mRNAs were evaluated in male and female HCC patients. There were no remarkable differences regarding the expression of miRNAs and the regulated mRNAs between male and female HCC patients (Supplementary Figures S1 and S2).

### 3.4. Analysis of CD4^+^ T Cells Infiltrates Correlation

**Figure 6:**
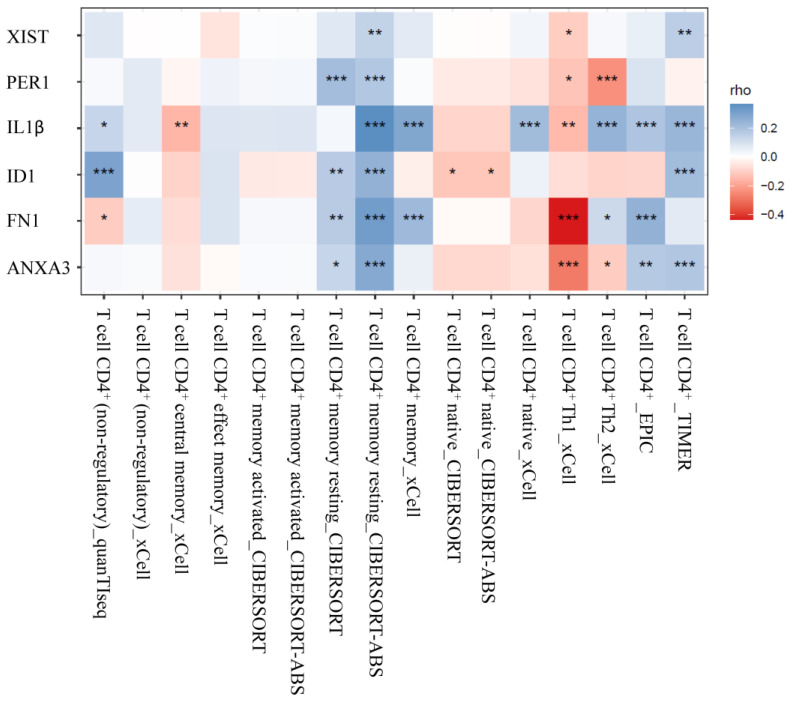
Analysis of CD4^+^ T cells infiltrates correlation. The correlation between the expression of miRNA-targeted genes and the level of CD4^+^ T cell infiltration in HCC. (* indicates *p* < 0.05; ** *p* < 0.01; and *** *p* < 0.001).

Using the TIMER2.0 website, we investigated the link between the expression of miRNA target genes and the amount of CD4^+^ T cell infiltration in HCC. The findings revealed a strong correlation between the expression of the miRNA target genes and the infiltration of total CD4^+^ T cells, particularly resting memory CD4^+^ T cells (Figure 6). Nevertheless, there was a negative correlation between the infiltration of Th1 CD4^+^ T cells and the expression of miRNA target genes. 

## 4. Discussion

In this investigation, we discovered circulating CD4^+^ T cells with differently expressed lncRNAs, miRNAs, and mRNAs in HCC patients. These transcripts are involved in the activation of numerous kinds of white blood cells, including myeloid leukocytes, neutrophils, and granulocytes. An XIST-related lncRNA–miRNA/mRNA network, which contains five miRNAs (hsa-miR-34a-5p, hsa-miR-369-3p, hsa-miR-494-3p, hsa-miR-495-3p, and hsa-miR-543) and five miRNA target genes (ID1, IL1β, ANXA3, PER1, and FN1), was rebuilt. This regulatory network associated with XIST may be positively connected with the infiltration of CD4^+^ T cells, particularly resting memory CD4^+^ T cells, but negatively correlated with Th1 CD4^+^ T cells. Our finding indicates that the regulation of circulating CD4^+^ T cells in HCC patients differs from that of healthy individuals.

XIST plays a role in each stage of X-chromosome inactivation. XIST recruits chromatin complexes that deposit heterochromatic alterations throughout the X, including H3K27me3 and ubiquitin-H2A, leading to transcriptional silence [27]. In SLE patients, XIST was predominantly up-regulated in activated T cell subsets and was associated with a more skewed X-chromosome allelic expression [28]. Mature nave T lymphocytes exhibit distributed patterns of XIST, but the inactive X lacks epigenetic changes. The activation of mature T cells restores the dormant X gene’s XIST transcripts and epigenetic changes [29]. The expression levels of XIST-related miRNAs and their regulated mRNAs did not change significantly between male and female HCC patients, despite the fact that XIST is a sex-linked lncRNA.

Regarding the XIST-related miRNAs found in this investigation, miR-34a was revealed to target 14 essential components of the NF-κB signaling pathway and to function as a major regulator of NF-κB in T cells [30]. The rise in miR-34a correlates with CD4^+^ and CD8^+^ T cell activation [30]. Targeting CXCR3, miR-34a-5p inhibits CXCR3 expression in CD4^+^ and CD8^+^ T cells. High levels of miR-34a-5p in naïve CD4^+^ T cells inhibit Th1 cell polarization by down-regulating CXCR3, leading to the diminished activation of cytotoxic T lymphocytes, natural killer cells, and natural killer T cells, as well as possible lymphopenia [31]. In addition, miR-369-3p regulates the inflammatory response as the signaling core. The up-regulation of miR-369-3p inhibits the LPS-induced inflammatory response; reduces the release of TNF, IL-6, IL-12, IL-1, and IL-1; and increases the production of anti-inflammatory cytokines, IL-10 and IL-1RA [32]. The nuclear translocation of NF-κB is likewise reduced by miR-369-3p [32]. In viral infections, inhibiting the expression of miR-369-3p dramatically reduces the expression of antiviral response genes, including CCL3, CCL5, CTSS, CXCL11, and IRF3 [33]. In addition, miR-494-3p has a function in macrophage polarization and activation via activating Wnt signaling to modulate M1/M2 polarization [34].

CD4^+^ T cells can exert their anti-tumor effects through various mechanisms. They can directly kill tumor cells by releasing cytotoxic molecules, such as perforin and granzymes. Additionally, they can activate other immune cells, such as CD8^+^ T cells and natural killer (NK) cells, which can further enhance the anti-tumor immune response [11,35]. CD4^+^ T cells also produce cytokines, such as IFN-γ and TNF-α, which have direct anti-tumor effects and can promote the recruitment and activation of other immune cells [11,35]. A decrease in CD4^+^ T cells is linked to a poor prognosis and a high rate of HCC recurrence [13]. In addition, the appearance of T cell types may play a crucial role in the development of HCC. For instance, Th1 cells are involved in anti-tumor immune responses and produce cytokines, such as IFN-γ, that activate other immune cells. HCC patients had a greater ratio of Th1 to CD4^+^ T cells than that in healthy controls, and patients with a higher tumor-infiltrating Th1 cell count have a considerably longer lifetime [36]. Moreover, Th17 cells have been associated with tumor progression and angiogenesis in HCC [37]. Tregs, on the other hand, have immunosuppressive properties and can dampen anti-tumor immune responses, promoting HCC growth [38,39]. Resting memory CD4^+^ T cells are a subset of CD4^+^ T cells that have encountered and responded to antigens in the past, leading to their differentiation into memory cells. These cells play a crucial role in mounting effective immune responses upon re-exposure to the same antigen. Several studies have suggested that the presence and activity of memory CD4^+^ T cells were identified as protective factors for the overall survival of HCC [40]. Immune cell analysis in the extracellular matrix of HCC showed that high-risk groups had lower cell fraction of memory CD4^+^ T cells than low-risk groups [41]. It has been observed that ID1-expressing CD4^+^ cells greatly boosted IL-2 production and NF-B activity in response to anti-CD3 stimulation in order to improve the proliferation and survival of naïve CD4^+^ cells [42]. IL-1 promotes the proliferation and development of antigen-specific CD4^+^ T cells toward a proinflammatory Th phenotype via the production of IFN-, IL-17, TNF-, and IL-4 [43,44]. Th1 cells have strong ANXA3 expression [36]. In the stress hormone signaling pathway, the elevation of PER1 expression inherently suppresses Th1 polarization in naïve CD4^+^ T cells [45]. Local manipulation of FN1 levels may be advantageous for boosting T cell concentration and is essential for T cell interstitial migration [46]. These cytokines may contribute to communication between immune cells and affect CD4^+^ T cell development. Despite indications suggesting these miRNAs are involved in the regulation of T cells, further research is required to determine the effect of the XIST-miRNAs/mRNAs network on the function of circulating CD4^+^ T cells in HCC.

## 5. Conclusions

In this work, the circulating CD4^+^ cells of HCC patients exhibited down-regulation of XIST, dysregulation of five XIST-related miRNAs, and up-regulation of five miRNA target genes, including ID1, IL1β, ANXA3, PER1, and FN1. The explored XIST-miRNA/mRNA regulation network revealed there are positive correlations in the infiltration of total CD4^+^ T cells, particularly resting memory CD4^+^ T cells, and negative correlations in the infiltration of Th1 CD4^+^ T cells.

## Figures and Tables

**Table 1:** Differential expression transcripts from NGS analysis in the patients with HCC compared with healthy controls.

Genes	Fold Change (log2)	P-adj	Chromosome	Type
RPS9	−7.13	0.0090	chr19	protein_coding
TAP2	−7.09	0.0104	chr6	protein_coding
LILRB1	−5.71	0.0020	chr19	protein_coding
XIST	−3.08	0.0001	chrX	lncRNA
SNORD3A	−2.95	0.0000	chr17	snoRNA
PCDH9	−2.76	0.0163	chr13	protein_coding
FCRL5	−2.15	0.0001	chr1	protein_coding
IGHM	−2.04	0.0006	chr14	IG_C_gene
GATA2	−1.98	0.0000	chr3	protein_coding
MS4A2	−1.89	0.0194	chr11	protein_coding
FCRL2	−1.73	0.0125	chr1	protein_coding
TCL1A	−1.70	0.0000	chr14	protein_coding
MS4A1	−1.45	0.0000	chr11	protein_coding
CD79A	−1.40	0.0442	chr19	protein_coding
LRRN3	−1.28	0.0079	chr7	protein_coding
LGALS12	−1.27	0.0471	chr11	protein_coding
FCRL1	−1.20	0.0389	chr1	protein_coding
PTPRS	−1.16	0.0156	chr19	protein_coding
RETN	−1.13	0.0021	chr19	protein_coding
AEBP1	−1.12	0.0184	chr7	protein_coding
U2AF1	1.03	0.0064	chr21	protein_coding
PER1	1.03	0.0000	chr17	protein_coding
IL1B	1.32	0.0000	chr2	protein_coding
G0S2	1.36	0.0119	chr1	protein_coding
RN7SL1	1.46	0.0061	chr14	misc_RNA
MIR222HG	1.64	0.0473	chrX	lncRNA
MAFF	2.15	0.0000	chr22	protein_coding
CEACAM8	2.54	0.0021	chr19	protein_coding
CTSG	2.67	0.0079	chr14	protein_coding
ID1	2.86	0.0000	chr20	protein_coding
ANXA3	2.89	0.0104	chr4	protein_coding
MMP8	3.03	0.0021	chr11	protein_coding
CAMP	3.15	0.0015	chr3	protein_coding
FN1	3.76	0.0471	chr2	protein_coding

**Table 2:** Differential expression of miRNAs from NGS analysis in HCC patients compared with healthy controls.

miRNAs	Fold Change (log2)	P-adj
hsa-miR-34a-5p	1.8277	2.96 × 10^−7^
hsa-miR-11400	−2.3323	5.06 × 10^−3^
hsa-miR-495-3p	−1.4377	8.24 × 10^−5^
hsa-miR-369-3p	−1.3807	4.40 × 10^−4^
hsa-miR-493-5p	−1.3633	1.75 × 10^−3^
hsa-miR-889-3p	−1.2994	3.01 × 10^−2^
hsa-miR-493-3p	−1.2379	6.44 × 10^−3^
hsa-miR-494-3p	−1.2308	7.77 × 10^−3^
hsa-miR-370-3p	−1.2273	1.10 × 10^−5^
hsa-miR-411-5p	−1.1649	3.01 × 10^−2^
hsa-miR-379-5p	−1.1397	2.53 × 10^−3^
hsa-miR-654-3p	−1.1235	6.53 × 10^−3^
hsa-miR-543	−1.0776	1.43 × 10^−3^

**Table 3:** miRNA–mRNA interaction predicted by ENCORI.

miRNA	Predicted Target Genes	Expression Data in the NGS Analysis
hsa-miR-34a-5p	PER1	Up-regulated
hsa-miR-34a-5p	IL1B	Up-regulated
hsa-miR-369-3p	MS4A1	Down-regulated
hsa-miR-369-3p	PCDH9	Down-regulated
hsa-miR-369-3p	ANXA3	Up-regulated
hsa-miR-370-3p	PER1	Up-regulated
hsa-miR-494-3p	MS4A1	Down-regulated
hsa-miR-494-3p	PCDH9	Down-regulated
hsa-miR-494-3p	ID1	Up-regulated
hsa-miR-495-3p	PCDH9	Down-regulated
hsa-miR-495-3p	IL1B	Up-regulated
hsa-miR-495-3p	ID1	Up-regulated
hsa-miR-543	PCDH9	Down-regulated
hsa-miR-543	FN1	Up-regulated
hsa-miR-543	GATA2	Down-regulated
hsa-miR-654-3p	MS4A1	Down-regulated
hsa-miR-654-3p	ID1	Up-regulated
hsa-miR-889-3p	LILRB1	Down-regulated
hsa-miR-889-3p	ID1	Up-regulated
hsa-miR-889-3p	GATA2	Down-regulated

**Table 4:** XIST-related miRNAs predicted by ENCORI.

miRNAs	LncRNA	Target Sequence
hsa-miR-34a-5p	XIST	gcugaCACAUACAUACACUGCCu
hsa-miR-369-3p	XIST	acauuuuugaaAAGUAUUAUu
hsa-miR-369-3p	XIST	accacccccugAUGUAUUAUu
hsa-miR-369-3p	XIST	uuAGAUUGA-CA-GUAUUAUg
hsa-miR-370-3p	XIST	ugaccacugcugggCAGCAGGa
hsa-miR-370-3p	XIST	gagagcugaguCUUCAGCAGGu
hsa-miR-370-3p	XIST	cuuucuUUUCCUCCCAGCAGGg
hsa-miR-370-3p	XIST	ccccuuUUGCAGUACAGCAGGg
hsa-miR-370-3p	XIST	cccuuuUUGCAGUACAGCAGGg
hsa-miR-370-3p	XIST	ugcccuUUGCAGUACAGCAGGg
hsa-miR-370-3p	XIST	cuuccuUUCCCUUCCAGCAGGg
hsa-miR-370-3p	XIST	ccccuuUUGCAGUACAGCAGGg
hsa-miR-370-3p	XIST	ucaccuUUCCCUUCCAGCAGGg
hsa-miR-370-3p	XIST	ccacuuUUCCCUUCCAGCAGGa
hsa-miR-370-3p	XIST	ccuccuuggCAAAGCAGCAGGa
hsa-miR-370-3p	XIST	uauuccUUUUGUCUUACAGCAGGg
hsa-miR-370-3p	XIST	cucccuUUGUAUUCCAGCAGGg
hsa-miR-370-3p	XIST	ccccauUUGCA-UUCAGCAGGg
hsa-miR-494-3p	XIST	cucugcaagguacUAUGUUUCg
hsa-miR-494-3p	XIST	ugucUUACCCAUUUCCAUGUUUCu
hsa-miR-495-3p	XIST	uaaAAGGGCUCAGGGUUUGUUc
hsa-miR-543	XIST	caaGACUGUCACUUGGAAUGUUc
hsa-miR-654-3p	XIST	--------------CAGACAUu
hsa-miR-654-3p	XIST	acuucccucaggAGCAGACAUu
hsa-miR-889-3p	XIST	cuucUGGUAGAGUGGGAUAUUAu
hsa-miR-889-3p	XIST	uuuuacagcaaggGAUAUUAa

## Data Availability

Data sharing is not applicable to this article.

## Supplementary Materials

The following supporting information can be downloaded at: https://www.mdpi.com/article/10.3390/biomedicines11071848/s1, Figure S1: The expression of XIST-related miRNAs in circulating CD4^+^ T cells of male and female patients with HCC versus healthy controls; Figure S2: The expression of the regulated mRNA target genes of XIST-related miRNAs in circulating CD4^+^ T cells of male and female patients with HCC versus healthy controls; Table S1: The clinical data of 20 people who participated in the NGS assessment of differential mRNA, lncRNA, and miRNA gene expression; Table S2: The clinical data of 160 individuals who had their transcript expression validated using real-time qPCR; Table S3: The primers sequences used in real-time quantitative polymerase chain reaction; Table S4: The target mRNAs of eight XIST-related miRNAs predicted by ENCORI.
