# Supplementary material for: Exploring the Regulatory Role of XIST-microRNAs/mRNA Network in Circulating CD4+ T Cells of Hepatocellular Carcinoma Patients
Source: Biomedicines. 2023 Jun 27;11(7):1848. doi: 10.3390/biomedicines11071848 (PMC10376435; doi:10.3390/biomedicines11071848)
Supplement: Supplementary file 1 [file biomedicines-11-01848-s001.zip › biomedicines-2479249-supplementary.pdf]

biomedicines

MDPI

Supplementary Table S1. The clinical data of 20 people who participated in the NGS assessment of differential mRNA, lncRNA, and miRNA gene expression.

	**Health (*n* = 10)**	**HCC (*n* = 10)**
Age (years, mean ± SD)	36.70±7.32	66.40±10.84
Gender		
Male, n (%)	1 (10%)	8 (80%)
Female, n (%)	9 (90%)	2 (20%)

Supplementary Table S2. The clinical data of 160 individuals who had their transcript expression validated using real-time qPCR.

	**Health (*n* = 100)**	**HCC (*n* = 60)**
Age (years, mean ± SD)	38.19±8.50	65.40±10.03
Gender		
Male, n (%)	24 (24%)	48 (80%)
Female, n (%)	76 (76%)	12 (20%)

Supplementary Table S3. The primers sequences used in real-time quantitative polymerase chain reaction.

**Targeted genes**	**Order**	**Sequences**
XIST	forward	5'-ACGCTG CATGTGTCCTTAG-3'
	reverse	5'-GAGCCT CTTATAAGCTGTTTG-3'
ID1	forward	5′-GCAGGTAAACGTGCTGCTCTACGA-3′
	reverse	5′-CGAGTTCAGCTCCAACTGAAGGTC-3′
IL-1β	forward	5′-TCCATGTCCTTTGTACAAGGAGAAGAAAGT-3′
	reverse	5′-TCGCTTTTCCATCTTCTTCTTTGGGTAAT-3′
ANXA3	forward	5′-ACTGAGATCCTGTGTTTAAGGAGCTT-3′
	reverse	5′-GGCTCGATGCAGTCTTTCGG-3′
PER1	forward	5′-GCCTCGGTACCAGCCATTCC -3′
	reverse	5′-GGTGTGTGCCGCGTAGT-3′
FN1	forward	5'-CCAGCCTACGGATGACTC-3'
	reverse	5'-AATGACCAC TGCCAAAGC-3'
